# Exploration of Quantum Chemistry Methods to Explain Mechanism of Mechanochemical Degradation of Typical Organic Pollutants

**DOI:** 10.3390/toxics13010023

**Published:** 2024-12-29

**Authors:** Xiaohui Zhang, Xiaoqian Xu, Zeya Zhang, Liang Pei, Tongshun Han

**Affiliations:** 1 Engineering Research Center of Coal-Based Ecological Carbon Sequestration Technology of the Ministry of Education, Key Laboratory of Graphene Forestry Application of National Forest and Grass Administration, Shanxi Datong University, Datong 037009, China; 17737136732@163.com (X.X.); 13967713095@163.com (Z.Z.); 16632816406@163.com (T.H.); 2 Xinjiang Institute of Ecology and Geography, Chinese Academy of Sciences, Urumqi 830011, China; 3 College of Resources and Environment, University of Chinese Academy of Sciences, Beijing 100049, China

**Keywords:** quantum chemistry, mechanochemistry, degradation pathway, organic pollutants

## Abstract

The high-efficiency ball milling treatment technology primarily combines the excitation of oxidation processes with high-speed physical collisions, thereby promoting the reaction processes and enhancing the degradation effectiveness of materials. This technology has gained widespread attention in recent years for its application in the degradation of organic solid chemical pollutants. In this study, quantum chemical density functional theory (DFT) was employed to first analyze the impact of electron addition and subtraction on molecular chemical bonds. The molecular energies of the target pollutants and their possible intermediates were then calculated, and the theoretical energies required for the degradation pathways of the target organic compounds under oxidative-enhanced ball milling were computed. This further validated the accuracy of the ball milling experimental results. The theoretical energy required for the complete mineralization of solid organic chemicals through ball milling degradation was calculated, with values of 16,730.74 kJ/mol for lindane, 20,162.46 kJ/mol for tetrabromobisphenol A, 10,628.04 kJ/mol for sulfamethoxazole, and 4867.99 kJ/mol for trimethoprim. By combining different ball milling experimental conditions, the theoretical reaction time required for the complete mineralization of the target organic chemicals can be calculated. The comparison of theoretical calculations with the experimental results provides new insights into the ball milling degradation process and degradation pathways of the target pollutants.

## 1. Introduction

Lindane (HCH), tetrabromobisphenol A (TBBPA), sulfonamides (SAs)—Compound Sulfamethoxazole Tablets (the main ingredients of which are sulfamethoxazole and trimethoprim, abbreviated as SMX and TMP, respectively) are typical organic chemicals used in everyday life and industrial production. The widespread use of these organic chemicals has led to serious environmental pollution problems [1,2,3], so it is essential to adopt efficient and feasible technology to deal with these chemicals. Mechanochemical techniques, due to their small size, versatility, and efficiency are commonly used in applied research for the treatment of organic pollutants. At present, domestic and foreign organic pollutant mechanochemical technology degradation reports, focusing on the operating parameters of the planetary ball mill, studied the ball milling model and energy changes and the changes in the structure of the material [4], and explored the mechanism of mechanochemical degradation of solid organic matter [5].

Quantum chemistry is a basic discipline that uses the basic principles and techniques of quantum mechanics to study chemical phenomena. By scrutinizing the nature of the molecular orbitals, the researchers delved into the energies and structures of the target molecules [6,7]. Using quantum chemical theory, one can theoretically approximate the chemical bond lengths of molecules and scrutinize the issues related to the molecular structure, properties, and interactions between molecules during material transformations. In recent years, many researchers have utilized quantum chemical density functional theory (DFT) to validate experimental results and thus gain a more complete understanding of the underlying chemical processes. Shanqing Li [8] used DFT to investigate the reaction mechanism of the conversion of chlorophenol compounds into dioxin-like compounds. Shouxian Zhong [9] used DFT to optimize the geometry of fluorinated pentacene molecules and performed frequency analysis to show that fluorinated pentacene molecules are organic semiconductor materials. Xiaowei Jiao [10] used DFT to study the degradation mechanism of liquid-phase sulfamethoxazole with -OH, and explored the effects of the metal ions, Ca^2+^, Mg^2+^, Cu^2+^, Zn^2+^, and Fe^3+^, on this degradation mechanism, respectively. Xinze Geng [11] investigated the mechanism of mechanochemical activation and bromine modification on the performance of fly ash demercury by combining experiment, characterization analysis, and DFT theoretical calculation. Yi Li [12] applied mechanochemical methods in the alkynyl ring isomerization reaction to synthesize bicyclic structural products via 1,6-alkynyl substrates, and revealed the reaction mechanism using DFT calculations. Ruoning Guo [13] investigated the mechanochemical degradation process of PFOA, constructed a mechanochemical reaction system for the defluorination of PFOA, and proposed a cyclic degradation pathway for PFOA by analyzing the obtained intermediates of PFOA degradation through DFT calculations and LCMS detection. Shouxi yu [14] utilized DFT theoretical calculations to explore the role of different oxygenate-containing acid radicals on the photodegradation performance of Dichlorvos. It is clear that the molecular properties, energy distribution, and reaction pathway can be predicted via quantum chemical calculations [15,16], and compared with the experimental data to verify the accuracy and consistency of the experimental results [17,18,19].

Commonly used computational theories in quantum chemistry include Hartree–Fock (HF) theory, Møller–Plesset perturbation theory (MP2-MP6), Gaussian 01, Gaussian 02, and Gaussian 03 methods, as well as density functional theory (DFT) [20]. Among them, density functional theory (DFT), which was developed on the basis of Thomas–Femri theory [21], is now widely used in the study of chemical reactions, describing the physical properties of the ground state of the system through particle density, especially for surface reactions, where DFT has an absolute advantage over other quantum chemical methods [22]. The latest DFT methods have been able to reach the level of Møller–Plesset perturbation theory in terms of accuracy, but the computational effort is only equivalent to that of the HF methods, and thus the Gaussian 03 method is still the preferred theory for calculating the electronic structure of molecular ground states.

In this paper, the DFT method is applied, on the one hand, to determine the optimal geometrical configuration of the molecules through optimization to keep the structure in the lowest energy state [23] to obtain the exact bond lengths of the molecules after the loss and gain of electrons, to study the effect of electron gain and loss on the bond lengths of the molecules, and to deduce the order of the chemical bond breaking, with a view to using it as a method to investigate the degradation pathways of organic solid chemicals and to identify the intermediates. On the other hand, by calculating the total energy, ionization energy, and electron affinity energy of the intermediates for the degradation of the target pollutants, and comparing the results of the ball milling experiments from the molecular point of view, the chemical reactions of typical organic pollutants such as lindane (HCH), brominated flame retardant (TBBPA), sulphamethoxazole (SMX), and trimethoprim (TMP) are discussed in an attempt to validate the accuracy of the degradation pathways by using the heats of reaction obtained from theoretical calculations to compare them with the experimental results.

## 2. Changes in Bond Length

Neutral molecules or the loss of neutral molecules by radicals in electron-generated negative ions results in the weakening of certain chemical bonds within the molecule, making them more prone to breakage [15]. Conversely, a decrease in bond length typically strengthens the chemical bond, enhancing its resistance to fracture [24,25]. Oxidation and reduction reactions are fundamentally characterized by the transfer of electrons. In oxidation, substances lose electrons, resulting in an increase in the oxidation number, while in reduction, substances gain electrons, leading to a decrease in the oxidation number. Thus, electron loss occurs in oxidation, and electron gain occurs in reduction [26]. Therefore, the study of the effects of the increment and decrement of electrons on the chemical bond lengths within pollutants can help to analyze the effects of chemical redox reactions on the aforementioned bonds, and thus elucidate the mechanisms and methods of chemical redox. In this section, Gaussian 03 is used to perform density functional theory (DFT) calculations and obtain the bond length information of the target molecules.

### 2.1. HCH

**Figure A1:**
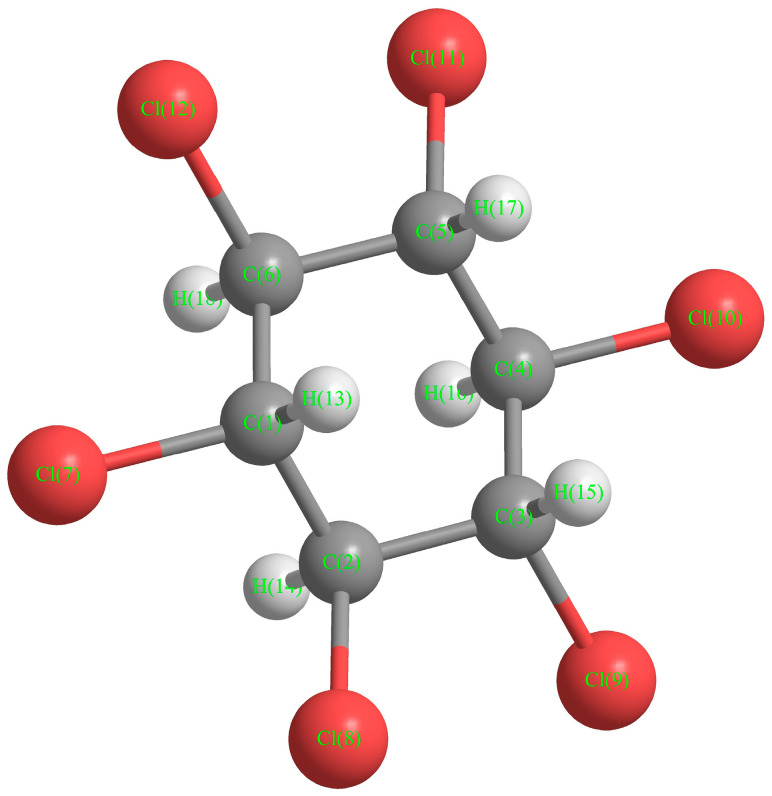
Molecular structure of lindane.

Lindane is 1, 2, 3, 4, 5, 6-hexachlorocyclohexane, has the chemical formula C_6_H_6_Cl_6_ [27], and the molecular structure is shown schematically in Figure A1. All the calculated bond lengths of lindane, including C-C, C-Cl and C-H bond lengths, are shown in Table 1. During the analysis in this paper, all the chemical bonds of lindane are identified with C atoms as the reference, e.g., with C1 as the reference, it means that the C-C bonds of C1 are identified sequentially along the direction of the numbering sequence, and the C-C bonds on C1 are identified as C1-C2 bonds, and the C-C bonds of C6 are identified as C6-C1 bonds.

Comparing Table 1, Table 2 and Table 3, the decrease in electrons causes the C-C and C-H bonds of lindane to become generally longer, while the C-Cl bonds become mostly shorter; the increase in electrons appears to have the opposite effect, causing the C-C and C-H bond lengths of lindane to increase or decrease differently, while the C-Cl bonds become mostly longer. Specifically, for chemical bonds at different positions, the effects of electron increase and decrease on bond lengths are also different. Comparing Table 1 and Table 2, it can be seen that the C6-C1 bond of C1-C6 is the only one that becomes slightly shorter by 0.000777 Å when decreasing by one electron, and the rest of the C-C bond lengths increase in the range of 0.004787–0.017575 Å. The C-Cl bond of C3 is the only one that becomes slightly longer by 0.005806 Å, and the rest of the C-Cl bond lengths decrease in the range of 0.000902–0.045396 Å range; the C-H bond of C4 is the only one that is slightly shorter by 0.001881 Å, and the rest of the C-H bond lengths increase in the 0.002366–0.006107 Å range. With the addition of one electron, the C-C bond lengths of C4 and C5 increase by 0.016076 Å and 0.005481 Å each, and the rest of the C-C bond lengths are shortened in the range of 0.002092–0.17828 Å. The C-Cl bond of C6 is the only one that is shortened by 0.015493 Å, and the C-Cl bond lengths of C1 and C2 increase by 0.961523 Å and 1.096456 Å for C1 and C2, with the rest of the C-Cl bond lengths ranging from 0.011856 Å to 0.02766 Å. The C-H bond lengths of C3 and C6 are increased by 0.001359 Å and 0.005604 Å each, with the rest of the C-H bond lengths shortened in the range of 0.000268 Å to 0.008662 Å.

The reduction reaction of lindane reveals that the C-Cl bonds at the C1 and C2 positions are prone to cleavage and dechlorination, while the C-H bond at the C6 position is more easily broken and dehydrogenated. In contrast, in the electronically reduced state of lindane during oxidation, the C-H bond is readily attacked and cleaved. This further supports the idea that lindane is easily dehydrogenated to hexachlorobenzene through reduction reactions mediated by free radicals. Additionally, C-C bond cleavage is more likely to occur, favoring benzene ring breakage during oxidation.

A comparison with the degradation pathway of lindane reported in the literature [28] shows that C-C bond cleavage does not occur at the beginning of the degradation process. This suggests that lindane is primarily susceptible to reduction initially, and that the R1 reaction is unlikely to occur. Quantum chemical calculations further indicate that the C-Cl bond at the C2 position is readily cleaved under oxidation, confirming that the R2 reaction of trichlorobenzene can undergo dechlorination and degradation through oxidation. Overall, the analysis of chemical bond length changes aligns well with the proposed degradation pathway for lindane, as suggested by the reported literature.

### 2.2. TBBPA

**Figure A2:**
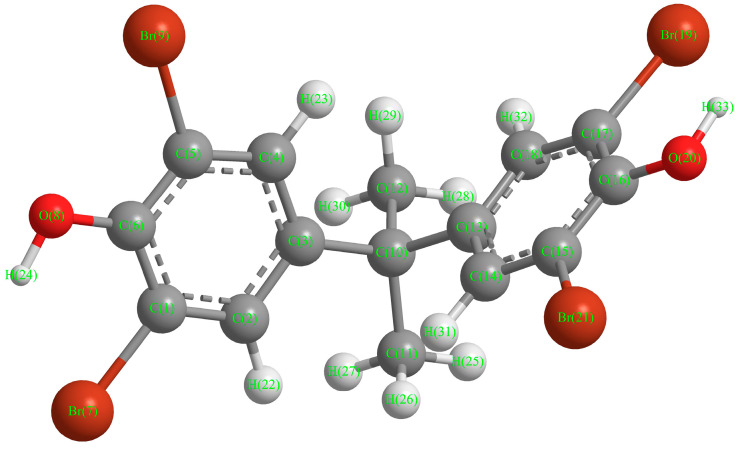
Molecular structure of TBBPA.

The molecular formula of tetrabromobisphenol A is C_15_H_12_Br_4_O_2_, and the molecular structure is shown in Figure A2. Table 4, Table 5 and Table 6 show the calculated bond lengths of TBBPA molecules with C atoms as reference markers.

Comparing Table 4 and Table 5, it can be seen that when electron reduction occurs, the C-O bond and C-Br bond on the benzene ring both become shorter, the C-H bonds all become longer in the range of 0.001866–0.002371 Å, and the C-C bond length increases and decreases, with an increase in the range of 0.006197–0.026797 Å and a decrease in the range of 0.003562–0.008145 Å; the C-C bond lengths of the C10-connected benzene rings are all reduced, with C10-C12 increasing by 0.001018 Å and C10-C11 increasing by 0.001655 Å, while the C-C bonds linking the heterocycles all become longer, with C3-C10 decreasing by 0.009534 Å and C13-C10 decreasing by 0.007206 Å.

When electrons are added, both the C-O and C-H bonds on the benzene ring lengthen, with the C-O bond increasing by 0.004436–0.009841 Å and the C-H bond increasing by 0.002614–0.006943 Å. The C-Br bond on the two benzene rings changes differently. On one benzene ring, the two C-Br bonds increase by 0.015874–0.808265 Å, while on the other ring, the two C-Br bonds decrease by 0.005294–0.008566 Å. It is noteworthy that the C-Br bond of C17 reaches a length of 2.732409 Å, which is presumed to have already undergone a tendency to fracture, and this inference corroborates the authors’ already reported literature [29] on the degradation pathway of TBBPA, where in the initial reaction, TBBPA’s C -Br bond is the most preferred site for decomposition [30], similar to other reports [31,32] of the debromination degradation of TBBPA in the liquid phase. The C-C bond lengths increase and decrease, with a small range of overall changes, except for a large decrease of 0.011993 Å for the C17-C18 bond lengths and an increase of 0.016595 Å for the C13-C18 bond on one benzene ring.

A comparison of the effect of electron gain and loss on the range of molecular bond length growth revealed that dehydrogenation and debromination reactions are more likely to occur on the benzene ring, the C-C bond on the hetero chain where C10 is attached to the benzene ring is susceptible to breaking, and TBBPA is also susceptible to reduction reactions, resulting in competing reactions during degradation, which is consistent with the oxidation and degradation pathways that have been identified in the studies of the oxidative effects of oxidizing agents, such as CuFe_2_O_4_ magnetic nanoparticles, hydroxyl radicals, and manganese oxides, on the oxidation and degradation of TBBPA degradation pathways identified in studies [33,34,35].

### 2.3. SMX

**Figure A3:**
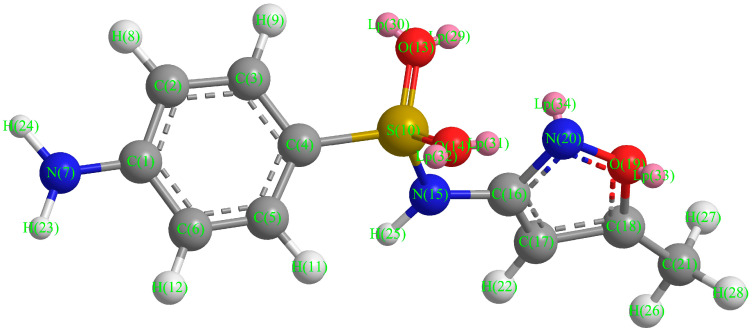
Molecular structure of SMX.

The molecular formula of sulfamethoxazole SMX is C_10_H_11_N_3_O_3_S. The structure is schematically shown in Figure A3. Table 7, Table 8 and Table 9 show the calculated bond lengths of all the chemical bonds of SMX.

Comparing Table 7, Table 8 and Table 9, it can be seen that the pattern of the effect of electron increase and decrease on the C-C bond is not very obvious, and the C-C bond length shows spaced lengthening and shortening, which is mainly due to the fact that the C-C bond is not the main target of the chemical action, while the other bonding sites will affect the change in the C-C bond length when they are attacked. Overall, the S-N bond is more affected when electrons increase and is most susceptible to attack and breakage, and it can be seen that the S10-N15 bond length has reached 6.218057 Å, indicating that breakage has occurred.

There are two situations when a radical attacks an S atom: one is to attack from the side of a six-membered aromatic ring, and the other is to attack from the side of a five-membered heterocycle. The high reaction energy barriers for both pathways may be caused by the large steric hindrance when the radical attacks the S atom. The C-N and N-O bonds also become longer when electrons are added, making them more likely to break, while the C-O bond shows the opposite trend. Studies have shown that SMX has two cleavage methods: S-N bond cleavage and C-S bond cleavage. Both reactions release heat energy. From a thermodynamic and kinetic point of view, the S-N bond cleavage reaction is more likely to occur. Both the S-N bond and the C-S bond have a strong tendency to cleave, and the S-N bond cleaves to a greater extent than the C-S bond, which is consistent with the lower reaction energy barrier for S-N bond cleavage than for C-S bond cleavage [10].

### 2.4. TMP

**Figure A4:**
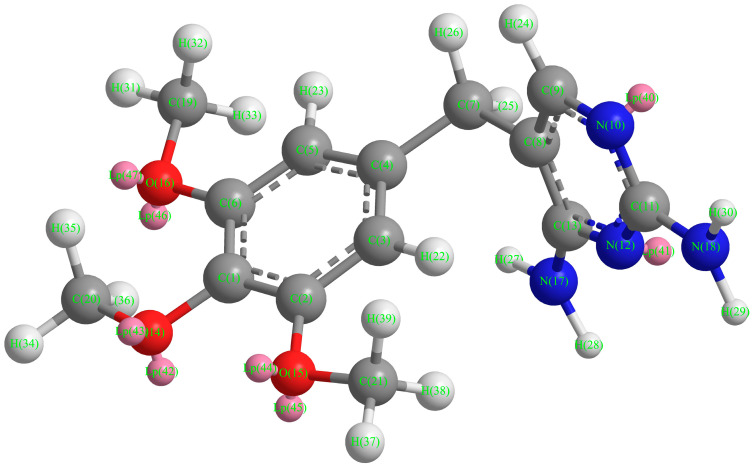
Molecular structure of TMP.

The structural formula of trimethoprim TMP is shown in Figure A4. Table 10, Table 11 and Table 12 list all the calculated bond lengths of TMP. Comparing Table 10, Table 11 and Table 12, it can be seen that when the electron is reduced, the C-C bond on the benzene ring does not change consistently. The C-C bond between C1-C2, C4-C5, and C6-C1 becomes longer, while the other C-C bond lengths become shorter.

Combining the shortening of the C-O bond length of C1/C2/C6 with the observation that the benzene ring is more likely to undergo ring opening and degradation when an oxidation reaction occurs, i.e., when electrons are reduced, it can be inferred that the C-C bond is weakened and the C-O bond is strengthened. The reduction in electrons does not have a significant effect on the C4-C7 and C7-C8 bonds that connect the two ring structures. The bond length of C8-C13 and C8-C9 becomes longer, indicating that this ring structure is more likely to break at the C-C bond when electrons are reduced.

## 3. Molecular Energy and Reaction Heat

Changes in molecular energy are a key determinant in analyzing the feasibility of material degradation. By analyzing the degradation process of the target pollutants, the total intermediate energy, ionization energy, and electron affinity energy were obtained. The ionization energy is the energy change of a molecule after the reduction in one electron, expressed as the energy difference between its cation and the neutral molecule; electron affinity energy is the energy change of a molecule after the addition of one electron, expressed as the energy difference between the neutral molecule and its anion [36]. Higher ionization energies indicate greater difficulty in losing electrons, which implies greater resistance to oxidation. Conversely, higher electron affinities indicate that it is easier to gain electrons and undergo reduction [37]. In this section, the energy required for the complete mineralization of the target pollutants is calculated from the molecular energy point of view, which is discussed in comparison with the experimental results. The total energy of the target pollutant molecules and their intermediates was calculated using B3LYP/6-31G (d, p), and the ionization and electron affinity energies were calculated using B3LYP/6-31+G (d), within the Gaussian 03 density functional theory (DFT) method.

### 3.1. HCH

The analysis of the energy differences calculated the energy changes of the intermediates produced during lindane degradation. Table A1 presents the total energy, ionization energy, and electron affinity values for lindane and its degradation products. Lindane is listed as the second structure in Table A1, with a total energy of −2993.397905 Hartree, an ionization energy of 9.660051 eV, and an electron affinity of −0.0661 eV. It can be observed that lindane’s total energy and electron affinity gradually decrease, while the change in ionization energy shows a weaker trend, first decreasing and then increasing. This suggests that lindane faces an initial challenge during the degradation process. The four structures (1, 3, 4, and 5) in the table have positive electron affinities, indicating that they are electron acceptors and are more susceptible to reduction reactions. In contrast, the remaining eight molecular structures have negative electron affinities, suggesting that the process of electron acquisition requires the absorption of energy. This observation is consistent with the degradation reaction pathway proposed in the literature [28]: structures 3, 4, and 5 are primarily intermediate products of dehydrogenation, which is a reduction process; structure 1 represents the first step in the oxidation reaction between the lindane molecule and a free radical, where the free radical replaces three hydrogen atoms, resulting in a positive electron affinity. The degradation pathways of the other molecular structures are primarily oxidation processes, which are associated with negative electron affinities.

In terms of the law of change, except for the simplest structures of benzene and phenol, the ionization energy generally decreases with the presumed degradation pathway; the law of change in electron affinity is relatively clear, and it also gradually decreases with the order of the degradation pathway. Therefore, combined with the analysis of the chemical bond length of lindane in the previous section, the gradual decrease in ionization energy with the degradation pathway indicates that lindane is most difficult to oxidize at the beginning of degradation, and when the C-Cl bond breaks, the intermediate product is more likely to lose electrons and be oxidized. And from the change in electron affinity energy, it can be seen that at a higher chlorine content, the intermediate product is prone to reduction, and as the dechlorination proceeds, the electron affinity energy gradually decreases and then becomes negative.

The analysis of ionization and electron affinity energies allows one to validate the reasoning of Figure 11 on lindane degradation in the published report by the authors [28]. The degradation of lindane via ball milling is divided into three stages, regarding which there are two possible pathways in the first stage, and the energy that may be required to calculate the complete degradation of lindane is shown in Table 13.

Judging from the two pathways in the first stage, 1, 3, 5 trichlorobenzene is also obtained from lindane degradation. The energy required for pathway I is 48.6 times higher than that for pathway II. It is speculated that pathway II is more likely to occur. This is consistent with the previous analysis results on chemical bond lengths and ionization energies. The experimental dosage of lindane in the above simulated soil was 1.85 × 10^−6^ mol, and the theoretical maximum energy required for the complete mineralization of lindane can be deduced to be 31.0 J. The ball milling power is 0.356 W [38], and the reaction time is 87.08 s; that is, if all the energy generated during the ball milling process is used for the degradation of lindane, theoretically, the energy requirement for the complete degradation of lindane can be met in 87.08 s. According to the literature [38], it takes 378 s to transfer energy to all the material at least once, which shows that the energy of a single collision is sufficient to cause a degradation reaction in the material between the grinding balls, but it takes a relatively long time to ball-mill the entire material.

**Figure 1:**
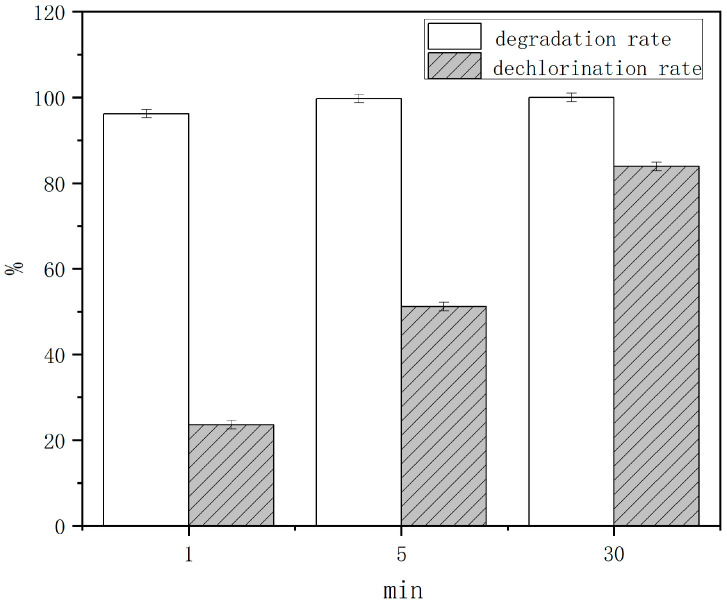
Degradation rate of lindane after different times of ball milling with the addition of NaOH.

In order to find out the ball milling time required to achieve the complete mineralization of lindane in contaminated soil, we carried out supplementary experiments on the reported literature [28], and the results are shown in Figure 1. The degradation rate of lindane can reflect its chemical bond breaking, and the chloride ion concentration can reflect the mineralization level of lindane to some extent. In the optimal process of sodium hydroxide-enhanced persulfate ball milling for the degradation of lindane in contaminated soil, lindane can reach a 100% degradation rate in 5 min, close to the theoretical calculation time of 378 s for all materials to achieve one full collision. The dechlorination of lindane at 5 min was only 51.5%, and the level of dechlorination at 30 min reached 83.9%, indicating that chlorinated organic matter was still present. The experimental results in Figures 3 and 4 of the literature [28] show that the dechlorination level reached 100% in 2 h. It can be seen that there is a certain gap between the theoretical calculations and experimental results. In addition to the effect of the molecular bond length changes in the second part, the possible reasons are as follows: pollutants in the soil environment, ball milling collision, and oxidative contact chances being relatively small compared to pure chemicals. Other organic matter in the soil environment will also affect the efficiency of lindane degradation, the degradation process cannot be continuous and smooth, which inevitably leads to the waste of ball milling energy, delaying the complete degradation of lindane in the soil time.

### 3.2. TBBPA

Table A2 shows that TBBPA has a high ionization energy and a low electron affinity, indicating that it is resistant to both oxidation and reduction, thereby confirming its persistence as a pollutant. The ionization energy of the intermediate products increases progressively, indicating that they become less susceptible to oxidative degradation. The electron affinities of the intermediate products are all positive, indicating that they are electron acceptors and more likely to undergo reduction reactions [39].

In comparison to Figure 7 in the authors’ published [29] report on the presumed degradation pathway of TBBPA, the total energy of the individual intermediates increases progressively along the degradation pathway. The energy required for the reaction was calculated based on the change in heat of the chemical reaction, as shown in Table 14. All the eight intermediate structures of TBBPA have positive electron affinity energies, indicating that they are electron acceptors and are more susceptible to the effects of reduction reactions. And the electron affinity energy decreases with decreasing molecular weight, indicating that their oxidizing properties decrease with decreasing molecular weight. This phenomenon is consistent with the change in reaction heat in the degradation pathway in Table A2. From the change in ionization energy, most of the TBBPA intermediate structures have ionization energies in the range of 4.08–7.77 eV, except for the structure of intermediate No. 5, which has an ionization energy as high as 16.86 eV. The ionization energy of TBBPA is very high, while the electron affinity energy is very low, which indicates that the TBBPA substance is difficult to be oxidized as well as reduced, and proves that it is a kind of difficult pollutant to be degraded. The ionization energy of the intermediate product shows a gradual increase, indicating that it is less and less easy to be oxidized and degraded.

It can be seen that TBBPA is completely mineralized, and the theoretical energy required is 20,162.46 kJ/mol. In the ball milling experiment, the number of TBBPA moles is 1.839 × 10^−4^ mol, and the energy required to achieve complete inorganic mineralization is 3707.23 J. The ball milling power is 0.356 W [38], and the reaction time can be obtained as 10,413.58 s. In other words, if all the energy generated during the ball milling process is used to degrade TBBPA, then 10,413.58 s is the time required to meet the energy requirements for the complete degradation of TBBPA. The time required to transfer energy at least once to all the material is 378 s, which shows that the energy of a single collision is not enough to cause a degradation reaction between the material and the grinding balls. The number of times that the material is ball milled must be at least 28, which is about 2.89 h. Compared with the degradation and debromination rates of TBBPA after 3 h of ball milling in Figure 1 of the literature experiment [29], they are basically close to the theoretical calculations.

### 3.3. SMX

As can be seen from Table A3, the ionization energy does not show a clear trend, indicating that the intermediate products of sulfonamides are not significantly more inclined to undergo oxidation. In terms of electron affinity, except for sulfonamides, which have a high electron affinity, substances 4, 5, and 6 all have negative values, and the electron affinities of the other substances are not very high, indicating that it is difficult for them to undergo reduction reactions. This shows that sulfa drugs are more likely to undergo reduction reactions, and the intermediate products further produced are relatively suitable for degradation via oxidation reactions, and the degree of oxidative degradation of the intermediate products does not gradually increase in the direction of the degradation pathway.

Compare the energy required for the reaction calculated in Figure 11 of the published report by the authors [40] regarding the proposed degradation pathway of SMX, as shown in Table 15. The ionization energy of SMX did not form a significant trend, indicating that the intermediate products of sulfonamide were not significantly more inclined to undergo oxidation reaction, while in terms of electron affinity energy, except for sulfonamide which had a higher electron affinity energy, the electron affinity energies of substances Nos. 4, 5, and 6 were all negative, and the electron affinity energies of the others were not very high, which indicated that it was difficult for them to undergo reduction reaction. This indicates that it is easier for sulfonamide to undergo reduction reaction, while the further intermediate products are relatively more suitable for degradation via oxidation reaction, and the degree of oxidative degradation of the intermediate products does not gradually increase in the direction of the degradation pathway.

SMX is completely mineralized, with a theoretical energy requirement of 10,628.04 kJ/mol. In the ball milling experiment, the molarity of SMX was 1.97 × 10^−3^ mol, and the energy required to achieve complete inorganic mineralization was 20,937.24 J, with a ball milling power of 0.356 W [38]. Theoretically, the reaction time for complete mineralization of SMX is 58,812.47 s. The time required to transfer energy at least once to all the material is 378 s. Therefore, the energy of a single collision is not sufficient to cause a degradation reaction of the material between the grinding balls. The number of times that the entire material must be ball milled is at least 156, which is about 16.34 h. Comparing this with the literature [40], under the optimal ball milling condition in the middle, it took only 2 h for SMX to reach 100% degradation, which indicates that the effect of zero-valent Fe-enhanced persulfate ball milling in degrading SMX was improved very significantly.

### 3.4. TMP

As can be seen from Table A4, the ionization energies of TMP and its 10 intermediates are all positive, indicating that the TMP molecule is relatively difficult to oxidize. A higher electron affinity indicates that it is more likely to receive electrons and be reduced. It can be seen that the electron affinities of TMP and its 10 intermediates are relatively low, and most of them are negative, indicating that they are also less likely to undergo reduction reactions. Therefore, it is shown that the TMP molecule is difficult to degrade under both oxidative and reductive conditions and is a relatively stable substance. Relatively speaking, the 11 substances are more likely to undergo reduction reactions.

Compare the energy required for the reaction calculated in Figure 12 of the published report by the authors [40] regarding the proposed degradation pathway of TMP, as shown in Table 16.

The electron affinity energies of TMP and its 10 intermediates are low and mostly negative, suggesting that it is also more difficult for reduction reactions to occur. The ionization energies of TMP and its 10 intermediates are all positive, suggesting that it is more difficult for oxidation reactions to occur in TMP molecules. It can be seen that it is difficult for the TMP molecule to be degraded under either oxidizing or reducing conditions, and it is a relatively stable substance, relatively speaking, so the 11 intermediates are more likely to undergo reduction reactions.

It can be seen from the calculation that the energy required for the complete mineralization of TMP is 4867.99 kJ/mol. In the experiment, the molar mass of TMP is 3.45 × 10^−4^ mol, and the total energy calculated is 1679.46 J. If the power of ball milling is introduced as 0.356 W [38], the theoretical reaction time required for the complete mineralization of TMP is 4717.57 s. The time required for all the material to transfer energy at least once is 378 s. Therefore, the energy of a single collision is not enough to cause a degradation reaction between the material and the grinding balls. The number of times that the TMP material is subjected to ball milling treatment must reach at least 13, which is about 1.31 h. Compared with the results in the literature [40], the degradation rate of SMX and TMP after 1.5 h of ball milling is close to 100%, which is close to the theoretical calculation.

## 4. Summary

This paper further verifies the degradation pathway of typical organic matter during high-efficiency ball milling treatment from the perspective of molecular structural changes, and discusses its degradation mechanism through quantum chemical calculation analysis. Chemical calculations show the following:

(1) By calculating the effect of electron gain and loss on the chemical bond lengths, the chemical bonds of the target pollutant molecular species atoms that are prone to breakage are analyzed and compared with the degradation pathways of the target organic pollutants in the results of the experimental studies, and a better correspondence is obtained. It can be seen that the method can play a certain auxiliary discriminatory effect for the identification of the degradation intermediates of the target organic pollutants similarly detected qualitatively via HPLC/MS or GC/MS.
(2) Calculating the electron affinity energy and ionization energy of the obtained intermediates and comparing the degradation pathways of the target organic pollutants with those of the experimental results further verified the accuracy of the exploration of the degradation mechanism.
(3) Based on the degradation pathways of the target organic pollutants in the experimental results, the theoretical degradation energies were as follows: lindane 16,730.74 kJ/mol, tetrabromobisphenol A 20,162.46 kJ/mol, sulfamethoxazole 10,628.04 kJ/mol, and trimethoprim 4867.99 kJ/mol. Corresponding to the optimal ball milling process of the experimental species, the theoretical reaction times required to achieve complete mineralization were 87.08 s for lindane, 2.89 h for tetrabromobisphenol A, 16.34 h for sulfamethoxazole, and 1.31 h for trimethoprim. Compared with the corresponding experimental reaction times, the theoretical calculations of tetrabromobisphenol A and trimethoprim were basically close to those of the experimental results, and the theoretical calculated values of lindane were much smaller than those of the experimental results, while those of sulfanilamide were much larger than those of the experimental results. The theoretical calculated value of lindane was much smaller than the experimental result, and the theoretical calculated value of sulfanilamide was much larger than the experimental result. Analyzing the reasons, lindane might have been dispersed in the simulated soil, which reduced the contact between lindane and oxidized radicals and the chance of collision with the ball, resulting in the prolongation of the reaction time required for the experiment. SMX, on the other hand, may have been subjected to enhanced oxidizing radicals, resulting in a much shorter reaction time.

In conclusion, the results of the ball milling experiments were verified via density functional theory calculations, the accuracy of experimentally derived degradation pathways was verified by comparative analysis from a quantum chemical perspective, the theoretical calculation of the theoretical energy required for the degradation of the target pollutant molecules was accomplished for the first time, and the theoretical time required for ball milling of the target pollutants was calculated by combining the ball milling modeling results with the experimental data, which further verified the experimental results’ credibility. Although there is a certain deviation between the theoretical calculation and the experimental results, it is believed that the application of this analytical perspective and method has a certain guiding value.

## Figures and Tables

**Table 1:** Chemical bond lengths of lindane (units: Å).

Chemical Bond	C1	C2	C3	C4	C5	C6
C-C	1.543592	1.543499	1.532538	1.532611	1.544064	1.544202
C-Cl	1.825455	1.834041	1.813599	1.801033	1.813069	1.834084
C-H	1.088049	1.087879	1.088977	1.095047	1.089056	1.087819

**Table 2:** Lindane chemical bond lengths with one electron removed (units: Å).

Chemical Bond	C1	C2	C3	C4	C5	C6
C-C	1.561167	1.554914	1.543803	1.549133	1.548851	1.543425
C-Cl	1.807829	1.799726	1.819405	1.779501	1.812167	1.788688
C-H	1.090415	1.092030	1.092768	1.093166	1.095163	1.093342

**Table 3:** Lindane chemical bond lengths with one electron added (units: Å).

Chemical Bond	C1	C2	C3	C4	C5	C6
C-C	1.365312	1.501268	1.530446	1.548687	1.549545	1.502851
C-Cl	2.786978	2.930497	1.841259	1.812889	1.825262	1.818591
C-H	1.079387	1.082334	1.090336	1.094131	1.088788	1.093423

**Table 4:** Chemical bond lengths of TBBPA (units: Å).

Chemical Bond	C1	C2	C3	C4	C5	C6
C-C	1.391948	1.396228	1.399859	1.384991	1.402708	1.398209
C-H/C-Br/C-O	1.909611	1.080330	/	1.082305	1.923728	1.349176
Chemical Bond	C14	C15	C16	C17	C18	C13
C-C	1.385503	1.403650	1.397395	1.391690	1.394451	1.401864
C-H/C-Br/C-O	1.082492	1.908971	1.349208	1.924144	1.080381	/
Chemical Bond	C3-C10	C13-C10	C10-C12	C10-C11		
C-C	1.542527	1.541649	1.547146	1.546724		

**Table 5:** TBBPA chemical bond lengths with one electron removed (units: Å).

Chemical Bond	C1	C2	C3	C4	C5	C6
C-C	1.388386	1.402425	1.421429	1.377870	1.416223	1.425006
C-H/C-Br/C-O	1.871314	1.082639	/	1.084331	1.893833	1.326333
Chemical Bond	C14	C15	C16	C17	C18	C13
C-C	1.379907	1.418743	1.422150	1.383545	1.408806	1.416432
C-H/C-Br/C-O	1.084358	1.879448	1.326872	1.890129	1.082752	/
Chemical Bond	C3-C10	C13-C10	C10-C12	C10-C11		
C-C	1.532993	1.534443	1.548164	1.548379		

**Table 6:** TBBPA chemical bond lengths with one electron added (units: Å).

Chemical Bond	C1	C2	C3	C4	C5	C6
C-C	1.394835	1.398138	1.403762	1.385940	1.404130	1.396130
C-H/C-Br/C-O	1.904317	1.082958	/	1.084919	1.915162	1.359017
Chemical Bond	C14	C15	C16	C17	C18	C13
C-C	1.394063	1.402063	1.391303	1.379697	1.411046	1.402428
C-H/C-Br/C-O	1.086009	1.924845	1.353644	2.732409	1.087324	/
Chemical Bond	C3-C10	C13-C10	C10-C12	C10-C11		
C-C	1.541143	1.542480	1.547855	1.549349		

**Table 7:** Chemical bond lengths of SMX (units: Å).

Chemical Bond	C1	C2	C3	C4	C5	C6
C-C	1.410387	1.386239	1.399980	1.398769	1.387566	1.409129
C-N/C-H/C-S	1.380953	1.086497	1.084704	1.779491	1.083277	1.086445
Chemical Bond	C16	C17	C18	O19	N7	N20
C-C/C-O/O-N/N-C	1.335616	1.505213	1.458715	1.442055		1.428726
C-H/C-C/N-H	/	1.078361	1.530952	/	1.026783	/
Chemical Bond	S10-N15	N15-C16	S10-O14	S10-O13	/	/
Bond Length	1.708900	1.399123	1.461244	1.462136	/	/

**Table 8:** SMX chemical bond lengths with one electron removed (units: Å).

Chemical Bond	C1	C2	C3	C4	C5	C6
C-C	1.418070	1.382633	1.407651	1.407940	1.381192	1.419520
C-N/C-H/C-S	1.358918	1.086489	1.085317	1.742566	1.085312	1.086530
Chemical Bond	C16	C17	C18	O19	N7	N20
C-C/C-O/O-N/N-C	1.406937	1.485129	1.472770	1.371969		1.350884
C-H/C-C/N-H	/	1.079995	1.533699	/	1.014718	/
Chemical Bond	S10-N15	N15-C16	S10-O14	S10-O13	/	/
Bond Length	1.821526	1.354701	1.460650	1.458478	/	/

**Table 9:** SMX chemical bond lengths with one electron added (units: Å).

Chemical Bond	C1	C2	C3	C4	C5	C6
C-C	1.403796	1.397606	1.396639	1.396199	1.397884	1.403371
C-N/C-H/C-S	1.418828	1.090574	1.087693	1.863824	1.087547	1.090552
Chemical Bond	C16	C17	C18	O19	N7	N20
C-C/C-O/O-N/N-C	1.416128	1.487863	1.461106	1.454649		1.439181
C-H/C-C/N-H	/	1.089223	1.523989	/	1.020123	/
Chemical Bond	S10-N15	N15-C16	S10-O14	S10-O13	/	/
Bond Length	6.218057	1.305934	1.539085	1.537975	/	/

**Table 10:** Chemical bond lengths of TMP (units: Å).

Chemical Bond	C1	C2	C3	C4	C5	C6
C-C	1.411813	1.396455	1.403557	1.393269	1.400142	1.399508
C-O/C-H/C-C	1.375649	1.365049	1.083727	1.516035	1.085337	1.373971
Chemical Bond	C19	C20	C21	C8	C13	N12
C-C/C-N/C-O	1.432063	1.430990	1.419795	1.417929	1.337431	1.343672
C-H/C-C/C-N	1.092005	1.092013	1.090847	1.518779	1.376035	/
	1.097555	1.094960	1.096912			
	1.092950	1.096544	1.097535			
Chemical Bond	C11	N10	C9	/	/	/
C-N/C-C	1.342990	1.340310	1.388854	/	/	/
C-N/C-H	1.371336	/	1.087786	/	/	/

**Table 11:** TMP chemical bond lengths with one electron removed (units: Å).

Chemical Bond	C1	C2	C3	C4	C5	C6
C-C	1.443176	1.395081	1.401222	1.416102	1.382510	1.432297
C-O/C-H/C-C	1.327330	1.344196	1.083560	1.516660	1.085352	1.355761
Chemical Bond	C19	C20	C21	C8	C13	N12
C-C/C-N/C-O	1.444449	1.448866	1.437598	1.434324	1.334594	1.341864
C-H/C-C/C-N	1.090162	1.089439	1.089384	1.511617	1.360593	/
	1.096022	1.090471	1.094904			
	1.092300	1.091008	1.094966			
Chemical Bond	C11	N10	C9	/	/	/
C-N/C-C	1.363566	1.321793	1.407259	/	/	/
C-N/C-H	1.345394	/	1.089853	/	/	/

**Table 12:** TMP chemical bond lengths with one electron added (units: Å).

Chemical Bond	C1	C2	C3	C4	C5	C6
C-C	1.406424	1.399491	1.421079	1.399937	1.395001	1.415615
C-O/C-H/C-C	1.386670	1.378114	1.084455	1.505383	1.087991	1.389848
Chemical Bond	C19	C20	C21	C8	C13	N12
C-C/C-N/C-O	1.429136	1.426888	1.418916	1.407477	1.357267	1.342547
C-H/C-C/C-N	1.092005	1.092013	1.090847	1.517966	1.397357	/
	1.097555	1.094960	1.096912			
	1.092950	1.096544	1.097535			
Chemical Bond	C11	N10	C9	/	/	/
C-N/C-C	1.331519	1.362437	1.400480	/	/	/
C-N/C-H	1.400918	/	1.085081	/	/	/

**Table 13:** Lindane degradation energy theoretical calculation table [28].

First Stage		Path I: 2 → 1 → 3 → 6	Path II: 2 → 6
Reaction Process	Reaction Heat (kJ/mol)	Calculation Formula	Parameter
ΔH _2→1→3→6_	8044.25	$\Delta H=E\left(2\right)-E\left(3\right)-3E\left(H_{2}O\right)-\frac{3}{2}E\left(Cl_{2}\right)$	$E\left(H_{2}\right)=-1.1785393\;\mathrm{Hrtree}$ $E\left({Cl}_{2}\right)=-920.3498845\;\mathrm{Hrtree}$ $E\left(HCl\right)=-460.8007767\;\mathrm{Hrtree}$ $E\left(O_{2}\right)=-150.2574266\;\mathrm{Hrtree}$ $E\left(CO_{2}\right)=-188.5809402\;\mathrm{Hrtree}$ $E\left(H_{2}O\right)=-76.4197366\;\mathrm{Hrtree}$
ΔH _2→6_	91.47	$\Delta H=E\left(2\right)-E\left(6\right)-3E\left(HCl\right)$	
Second Stage		6 → 7 → 8 → 9 → 10 → 11 → 12	
ΔH _6→12_	4599.81	$\Delta H=E\left(6\right)-E\left(12\right)-\frac{3}{2}E\left(Cl_{2}\right)$	
Third Stage		Mineralization Generates CO_2_ and H_2_O	
ΔH _mineralization_	4086.68	$\Delta H=E\left(12\right)+\frac{15}{2}E\left(O_{2}\right)-6E\left(CO_{2}\right)-3E\left(H_{2}O\right)\;\;\;\;\;\;\;\;$	
ΔHtotal I		16,730.74	
ΔHtotal II		8777.96	

**Table 14:** Theoretical calculation table for the energy required for TBBPA degradation [29].

Reaction Process	Reaction Heat (kJ/mol)	Calculation Formula	Parameter
ΔH _1→2_	−24,909.91	$\Delta H=E\left(1\right)-E\left(2\right)-0.5E\left(H_{2}\right)$	$E\left(H_{2}\right)=-1.1785393\;\mathrm{Hrtree}$ $E\left(O_{2}\right)=-150.2574266\;\mathrm{Hrtree}$ $E\left(Br_{2}\right)=-5143.3991121\;\mathrm{Hrtree}$ $E\left(CO_{2}\right)=-188.5809402\;\mathrm{Hrtree}\;$ $E\left(H_{2}O\right)=-76.4197366\;\mathrm{Hrtree}$
ΔH _1→3_	−26,318.63	$\Delta H=E\left(1\right)-E\left(3\right)-0.5E\left(O_{2}\right)$	
ΔH _1→4_	1,481,045.58	$\Delta H=E\left(1\right)-E\left(4\right)-0.5E\left(Br_{2}\right)$	
ΔH _1→5,8_	−22,908.92	$\Delta H=E\left(1\right)-E\left(5\right)-E\left(8\right)$	
ΔH _5→6_	1139.43	$\Delta H=E\left(5\right)-E\left(6\right)-0.5E\left(H_{2}\right)$	
ΔH _5→8_	3161.70	$\Delta H=E\left(5\right)-E\left(8\right)-0.5E\left({C_{3}H}_{6}\right)$	
ΔH _6→7_	1564.13	$\Delta H=E\left(6\right)-E\left(7\right)-0.5E\left(Br_{2}\right)$	
ΔH _8→9_	1556.54	$\Delta H=E\left(8\right)-E\left(9\right)-0.5E\left(Br_{2}\right)$	
ΔH _9→Phenol_	375.63	$\Delta H=E\left(9\right)-E\left(C_{6}H_{5}\mathrm{OH}\right)\;-0.5E\left(Br_{2}\right)$	
ΔH _Phenol→Benzene_	−235.43	$\Delta H=E\left(C_{6}H_{5}\mathrm{OH}\right)-E\left({C_{6}H}_{6}\right)\;-0.5E\left(O_{2}\right)$	
ΔH _Phenylene mineralization_	2571.03	$\Delta H=E\left({C_{6}H}_{6}\right)+5E\left(O_{2}\right)-\;3E\left(CO_{2}\right)-4E\left(H_{2}O\right)$	
ΔH _Benzene mineralization_	406.68	$\Delta H=E\left(12\right)+\frac{15}{2}E\left(O_{2}\right)-6E\left(CO_{2}\right)-3E\left(H_{2}O\right)$	
ΔH _total_	20,162.46		

**Table 15:** Theoretical calculation table of energy required for SMX degradation [40].

Reaction Process	Reaction Heat (kJ/mol)	Calculation Formula	Parameter
ΔH _1→2_	2853.34	$\Delta H=E\left(1\right)-E\left(2\right)-E\left(3\right)$	$E\left(H_{2}\right)=-1.1785393\;\mathrm{Hrtree}$ $E\left(N_{2}\right)=-108.9439495\;\mathrm{Hrtree}$ $E\left(O_{2}\right)=-150.2574266\;\mathrm{Hrtree}$ $E\left(CO_{2}\right)=-188.5809402\;\mathrm{Hrtree}\;E\left(H_{2}O\right)=-76.4197366\;\mathrm{Hrtree}$
ΔH _2→6_	2067.35	$\;\Delta H=E\left(2\right)+0.5E\left(H_{2}\right)+E\left(O_{2}\right)-E\left(6\right)-E\left(H_{2}SO_{4}\right)$	
ΔH _6→Benzene_	1620.66	$\Delta H=E\left(6\right)-E\left(C_{6}H_{6}\right)-0.5E\left(N_{2}\right)-E\left(H_{2}\right)$	
ΔH _Benzene mineralization_	4086.68	$\Delta H=E\left(12\right)+\frac{15}{2}E\left(O_{2}\right)-6E\left(CO_{2}\right)-3E\left(H_{2}O\right)$	
ΔH _total_	10,628.04		

**Table 16:** Theoretical calculation table for the energy required for TMP degradation [40].

Reaction Process	Reaction Heat (kJ/mol)	Calculation Formula	Parameter
ΔH _3→6,7_	698.37	$\Delta H=E\left(3\right)+2E(O_{2})-E\left(6\right)-E\left(7\right)-E(CO_{2})$	$E\left(H_{2}\right)=-1.1785393\;\mathrm{Hrtree}$ $E\left(N_{2}\right)=-108.9439495\;\mathrm{Hrtree}$ $E\left(O_{2}\right)=-150.2574266\;\mathrm{Hrtree}$ $E\left(CO_{2}\right)=-188.5809402\;\mathrm{Hrtree}\;E\left(H_{2}O\right)=-76.4197366\;\mathrm{Hrtree}$
ΔH _6→Phenol_	−1,472,428.40	$\Delta H=E\left(6\right)+\frac{15}{2}E(O_{2})-E\left(C_{6}H_{5}OH\right)-3E\left(CO_{2}\right)-\frac{9}{2}E\left(H_{2}O\right)$	
ΔH _Phenol→Benzene_	−235.43	$\Delta H=E\left(C_{6}H_{5}OH\right)-E\left(C_{6}H_{6}\right)-0.5E\left(O_{2}\right)$	
ΔH _Benzene mineralization_	4086.68	$\Delta H=E\left(12\right)+\frac{15}{2}E\left(O_{2}\right)-6E\left(CO_{2}\right)-3E\left(H_{2}O\right)$	
ΔH _7 Mineralization_	83.94	$\Delta H=E\left(7\right)+5E\left(O_{2}\right)-4E\left(CO_{2}\right)-3E\left(H_{2}O\right)-2E\left(N_{2}\right)$	
ΔH _total_	4867.99		

## Data Availability

Data are contained within the article and the appendixs.

## Appendix B

**Table A1:** Comparative energy analysis of lindane and intermediate products.

NO.	Molecular Formula	Total Energy (Hrtree)	Ionization Energy (eV)	Electronic Affinity (eV)
1		−3219.047954	9.950384	0.523592
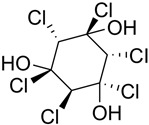
2		−2993.397905	9.660051	−0.0661
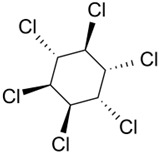
3		−2989.767444	9.082084	1.716961
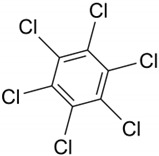
4		−2532.598758	9.576614	0.705967
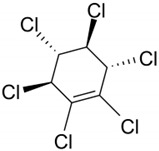
5		−2071.799085	8.895786	1.632986
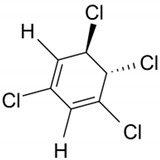
6		−1611.030415	9.325517	−0.05611
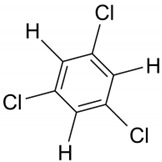
7		−1226.659314	8.844136	−0.35207
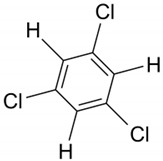
8		−842.2876064	8.477724	−0.82956
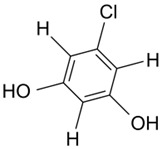
9		−457.9152132	8.229249	−0.70907
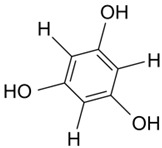
10		−382.6953839	8.188117	−0.82385
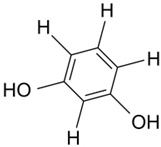
11		−307.4759719	8.421032	−1.23313
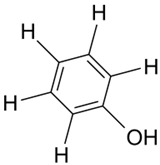
12		−232.257587	9.139659	−1.41723
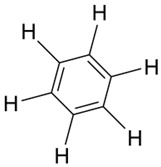

**Table A2:** Comparative energy analysis of TBBPA and intermediate products.

NO.	Molecular Formula	Total Energy (Hrtree)	Ionization Energy (eV)	Electronic Affinity (eV)
1		−11,026.0440961	275.176785	−266.512318
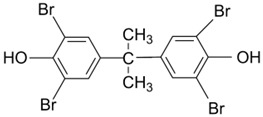
2		−11,015.966999	4.80554673	3.88259003
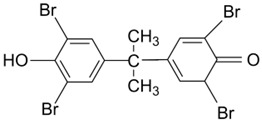
3		−10,940.8911479	5.30341414	2.88892393
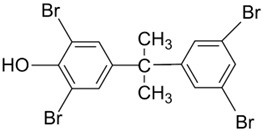
4		−8445.0016785	5.81977754	2.07080505
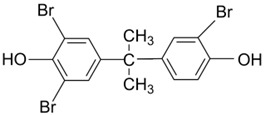
5		−5567.6347284	16.8567036	0.96780982
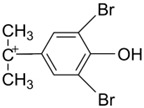
6		−5567.479452	6.49345077	1.61597791
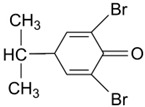
7		−2996.3756586	7.14183661	0.62101069
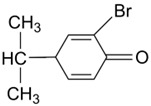
8		−5449.6836096	7.28859052	1.10851272
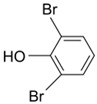
9		−2878.5769144	7.77235797	0.07284616
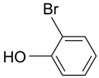

**Table A3:** Comparative energy analysis of SMX and intermediate products.

NO.	Molecular Formula	Total Energy (Hrtree)	Ionization Energy (eV)	Electronic Affinity (eV)
1		−1176.8768454	6.62403055	1.87138317
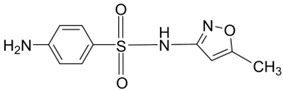
2		−836.1359587	8.06826931	0.30751795
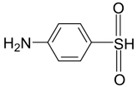
3		−341.8276843	6.07016344	0.62204233
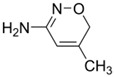
4		−891.4703636	7.79545686	−0.31096128
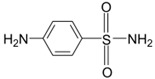
5		−911.3696233	8.09736205	−0.28036056
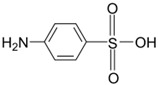
6		−287.5767125	7.44180373	−1.08851419
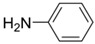
7		−362.7236502	8.93656826	0.74412403
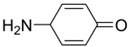

**Table A4:** Comparative energy analysis of TMP and intermediate products.

NO.	Molecular Formula	Total Energy (Hrtree)	Ionization Energy (eV)	Electronic Affinity (eV)
1		−1178.5478191	7.123794	−0.49611
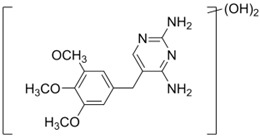
2		−1062.9497494	7.399306	0.05425
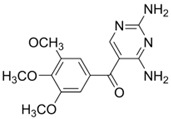
3		−989.0153056	9.436336	−2.80773
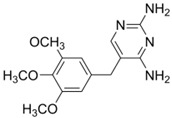
4		−988.9199379	7.211204	−0.45212
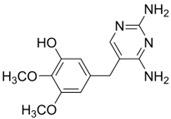
5		−1028.2228506	7.091024	−0.55431
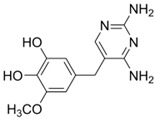
6		−651.003733	7.945348	−0.80285
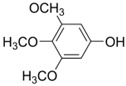
7		−450.2153288	7.159445	−0.38929
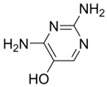
8		−563.4890644	7.555219	0.095224
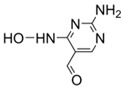
9		−489.5389619	7.157903	−0.5948
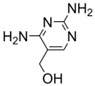
10		−488.3561876	8.035049	−0.03008
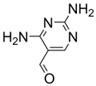
11		−342.3363269	9.476485	0.89395
